# The philosophical foundations of ‘health for all’ and Universal Health Coverage

**DOI:** 10.1186/s12939-022-01780-8

**Published:** 2022-11-05

**Authors:** Luke N. Allen

**Affiliations:** London School of Hygiene & Tropical Medicine, Keppel St, London, WC1E 7HT UK

**Keywords:** Equity, Inequalities, Ethics, Social justice, Proportionate Universalism

## Abstract

The WHO constitution calls for ‘health for all’ and Universal Health Coverage has been called “the ultimate expression of fairness”, however it is not always clear how health systems can move towards equity. Should we prioritise the needs of the worst off? And if so, should we direct resources to these marginalised groups or marginalised individuals? This article provides an overview of the philosophical underpinnings of health equity and proportionate universalism, highlighting the trade-offs involved in operationalising a core tenant of global health practice.

## A lofty aspiration

Health inequalities are ubiquitous [1–3]. Some arise from natural human variation and physiological differences, for instance people with white skin are more likely to develop skin cancer than people with black skin [4]. However, many other inequalities stem from avoidable and unfair social structures—such as the differences in all-cause mortality according to skin colour [5]. The inverse care law states that the supply of medical care is inversely proportionate to need [6], and the most disadvantaged groups in society almost universally experience the worst health outcomes [7]. WHO state that “many of the populations that have the worst health statuses face systemic discrimination based on race, ethnicity, gender, sexual orientation, socioeconomic status, location, religion, educational status and disability [8].

Addressing unjust inequalities is a fundamental tenet of global public health: the 1948 WHO constitution is built around the aspiration of ‘health for all’ [9] and the Alma-Ata and Astana Declarations on Primary Health Care espouse the principles of social justice and the ‘fundamental right to health without distinction of any kind’ [10, 11]. These principles were driving themes under the visionary leadership of Halfdan Mahler, who served three terms as WHO Director General from 1973 – 1988. During his tenure Mahler oversaw a major shift in focus from single diseases viewed through ‘medically tainted glasses’ to holistic primary health care and engagement with the wider social, political, and economic determinants of health [12]. He was instrumental in developing and leading the WHO’s defining ‘Health For All by 2000’ programme of work, seeking “a level of health that will permit all the people of the world to lead socially and economically satisfying and productive lives…based on the fundamental values of social justice and equity.” [13].

Universal Health Coverage (UHC) is the contemporary manifestation of health for all, and all WHO member states have committed to “achieve UHC, including financial risk protection, access to quality essential health-care services and access to safe, effective, quality and affordable essential medicines and vaccines for all” in Sustainable Development Goal target 3.8 [14].

But what do we actually mean by advancing health *for all*, and how might we get there – or at least begin moving in the right direction? This short review summarises the most important ethical theories that have undergirded attempts to operationalise this audacious concept in the form of Universal Health Coverage.

## Should we tackle inequalities?

Some economists and philosophers have argued that efforts to reduce inequalities are illiberal [15], unmeritocratic [16], and – in the view of Friedrich Nietzsche – reflective of moral failure [17]. Whilst these views are extreme, most philosophers and economists agree that a degree of inequality is socially desirable because it provides incentives for people to take personal responsibility for their actions [18]. The precondition for this inequality is a form of effort-based meritocracy where gains, success, and outcomes are related to skill and hard work – rather than parentage, private education, or social class. In other words, everyone should be able to achieve the same gains with the same effort. As Aristotle put it; “equals should be treated equally” [19].

Of course, in real life the playing field is not fair, and authors like Daniel Markovits has argued that meritocracy is a “pretence, constructed to rationalize an unjust distribution of advantage” [20]. Public anger at differential access to education, resources, and opportunities has manifest regularly throughout human history – including in contemporary demonstrations against the ‘one-percent’ moneyed elite [21].

Assuming that at least some health inequalities are unjust and should be tackled, there is a surprisingly broad spectrum of philosophical positions that can support the common goal of reducing inequalities. The three main schools of thought that have been developed to consider the distribution of social resources are *egalitarianism, sufficientism*, and *prioritarianism*. We will consider each in turn.

Egalitarian approaches concerned with equality. The primarily aim is to *close gaps* so that all people experience the same outcomes. In mathematical terms, the focus is on the range rather than the mean i.e. it doesn’t matter what the absolute outcome is, as long as everyone has the same. This can apply to inputs, outputs, or outcomes, leading to radically different policy goals e.g. ‘everyone has equal access to the same services’ vs ‘everyone achieves the same life expectancy’. Ideally, those with the worst baseline health outcomes would see their health improved to match the best-off, however proponents of egalitarianism can also implicitly or explicitly achieve their ends by ‘levelling down’ i.e. taking resources away from advantaged members of society. Most would agree that taking resources away from people so that everyone has nothing is perfectly equal, but probably undesirable. Efforts to reduce inequalities should ideally consider the absolute level of the given outcome, as well as the relative distribution.

In contrast to egalitarians, proponents of sufficientism take the view that inequalities can largely be ignored as long as everyone *has enough *[22]. The threshold for ‘enough’ can be couched in absolute terms, such as the US$1.90 international poverty line [23], or it might be a relative threshold, for instance Adam Smith famously argued that everyone should have enough to be able “to appear in public without shame” [24]. Similarly, the women’s suffrage demand for ‘bread and roses’ was an assertion that basic necessities extend beyond food and shelter to include education, art and beauty [25]. However it is defined, the definition of *enough* is commonly tied to evolving social standards. For instance, mobile phone ownership and an internet connection are basic necessities for participation in everyday life today but were opulent curiosities in the 1990s. Whilst sufficientism guarantees that everyone obtains a certain level, the focus is on the floor rather than the upper limits and aspirations of what a society can achieve.

The third main approach to addressing inequalities is prioritarianism [26]. Its proponents place primacy on the conditions of the worst-off members of society and judge the moral value of any action by the extent to which it improves their lot. Like sufficientists, prioritarians are not actually concerned with inequality in itself: they are only concerned with the inequitable distribution of resources and outcomes insofar as redistributing them would improve the status of the most disadvantaged. This can lead to acceptance of inequalities when there are no further actions that would change the status quo.

## Application to health inequalities

These three theories apply to inequalities in access to all forms of resources. For health inequalities it is important to make the distinction between inequalities stemming from immutable factors (e.g. skin colour), unjust social structures (e.g. institutional sexism) and outcomes over which people exercise a degree of personal agency, such as diet. It is important to recognise that there is a spectrum here, as ‘choices’ are heavily shaped and constrained by our environment [27].

Whitehead and Dahlgren have argued that inequalities become inequities when they are “unavoidable, unnecessary, and unfair” [28]. Michael Marmot goes on to say that “putting them right is a matter of social justice.” [1]. This position is ascendant within global health and aligns with elements of John Rawls’ theory of justice [29]. Rawls deftly combined the optimum level of inequality with a prioritarian approach using his ‘difference principle’; that inequalities are permitted insofar as they benefit the least advantaged in society, and his ‘maximin rule’; that interventions should be weighed by the extent to which they maximise the utility of the worst off. Together these principles only permit inequalities that would make the most disadvantaged even worse off if they were addressed [29].

Rawls’s theory of justice was confined to sovereign states and dealt with the distribution of services rather than health itself: he was not concerned with the pattern of health outcomes as long as the basic structure of society is just [29, 30]. However, Normal Daniels has argued that by demanding fair equality of opportunity, Rawls’s theory of justice requires a robust flattening of the socioeconomic health gradient [31, 32]. Both philosophers have been criticised for focusing on means and resources whilst implicitly disregarding human diversity and differing capabilities to use resources that leads to differences in outcomes [33].

Building on Aristotelian ethics [34] and Sen’s capability approach [27, 35], Ruger has argued that the concept of global health equity should focus on realising each individual’s capability to be healthy and function as a flourishing member of society [36, 37]. Her approach treats health as an instrumental and intrinsic good. Rather than pursuing the achievement of equal health outcomes, Ruger’s conceptualisation of ‘health for all’ centres on providing the social conditions required for people to have the capability to experience good health. She outlines four key domains: the quality of services and resources; personal capacity to enable healthy functioning; social support for health agency to allow individuals to make use of resources; and prevailing health norms [38].

## Operationalising ‘health for all’ with Universal Health Coverage

When we come back to consider WHO’s foundational aim of achieving the highest standard of health for all – without distinction, we can see that; 1) a highly aspirational, absolute threshold is being advanced; and 2) there is a concern for understanding and addressing differential attainment of that goal. The advent of Universal Health Coverage (UHC) – dubbed “the ultimate expression of fairness” by former Director General Margaret Chan [39]—helped to translate the lofty vision into the concrete aims of extending health services and financial risk protection. Whereas Mahler’s tenure highlighted the plight of the poor [40], the conceptualisation of UHC that was advanced under Chan’s leadership was built on a philosophical foundation of sufficientism: each country should select a minimum basket of services and a maximum financial exposure threshold that should be applied to every citizen [41].

Given that access is not universal for most services, UHC forces policymakers to consider which groups to include first as new services are rolled out. From the point of view of a health programme manager faced with suboptimal service coverage, their main concern may be to boost coverage rates as cost-effectively as possible with little regard for which group receives extended access first.

There is nothing intrinsically prioritarian in the definition of UHC, and concerns have been raised that “people who are poor could well gain little until the final stages of the transition from advocacy to achievement” [42]. In response to this perceived risk, WHO convened the C*ommission on Making Fair Choices on the Path to UHC*. The commissioners’ final report argued that “it is unacceptable to expand coverage for well-off groups before doing so for worse-off groups when the costs and benefits are not vastly different” [43]. In an accompanying editorial, Chan explained that “To include more people fairly, countries should first expand coverage for low-income groups, rural populations, and other groups disadvantaged in terms of service coverage, health, or both” [39]. This view echoes an open Lancet letter signed by 267 economists who argued stated that policymakers should focus on extending services to the “poorest and most marginalised populations.” [44].

Interestingly, whilst Rawls argued that the focus on the worst-off should be absolute, the WHO position tacitly implies that there is a threshold at which the additional costs of prioritising disadvantaged groups become unjustifiable. Another important but undefined issue is how to select which groups to target. The WHO equity consultive group has suggested nine core domains, based on earlier work by the *Commission on Social Determinants of Health*. These are income, wealth, education, occupation, ethnicity/race/indigeneity, gender, area of living (urban/rural), refugee/immigrant status, religious and political beliefs, and sexual orientation [43]. However, WHO does not seem to have adopted these domains in any further normative guidance.

## Universalism, selectivism, and the distribution of care

The idea of prioritising certain sociodemographic groups represents a marked departure from Beveridgean ‘general universalism’ – an impartial approach to welfare that does not take need into account when distributing social benefits. In Beveridge’s original – pointedly egalitarian—vision for the British NHS, everyone would be eligible and everyone would receive the same service, irrespective of sociodemographic characteristics, means, or need [45–47].

Systems based on the related principle of ‘specific universalism’ also seek to be impartial in the distribution of benefits, but they recognise that some social groups face barriers. In response, benefits are distributed within a framework of extending social rights, such as the right to health, as a way of ensuring that that services are genuinely available to all [48].

Carey, Crammond and De Leeuw have noted that both forms of universalism tend to conflate equality with equity, commonly leading to situations where those on the margins of society do not actually have their needs met [47]. As such, many governments have introduced elements of ‘selectivism’ to target the provision of services according to need across the social gradient.

The WHO report discussed above advocates for what is known as ‘positive selectivism’ – using membership of a social group to determine access, irrespective of the unique needs of individuals within those groups [43]. An alternative approach is ‘negative selectivism’ which uses means-testing to target individuals, irrespective of their sociodemographic characteristics [49]. Perhaps counterintuitively, negative selectivism has been repeatedly associated with poor outcomes, summarised by Francis-Oliveiero as “stigmatisation, increased social distance between recipients and non-recipients, administrative cost for means-testing, and also misclassifications, under-coverages and leakages” [50].

## Proportionate universalism

Aiming to find a balance between universalism and selectivism, Théda Skocpol proposed ‘targeted universalism’ in the early 1990s [51]. Her approach resonates with the ‘weighted priority’ form of prioritarianism that emerged in the late 1990s, and shifted from exclusively focusing on the worst-off towards distributing benefits to all, in accordance with baseline wellbeing [52, 53]. These ideas were adopted and adapted for public health by Michael Marmot who advocated for ‘proportionate universalism’ in his 2010 report *Fair Society, Healthy Lives *[1]. Proportionate universalism combines positive selectivism with universalist principles of equality and fairness; seeking to provide services to all, with additional resources provided to members of specific groups in order to offset the structural challenges that they face: “actions should be universal, but with an intensity and a scale that is proportionate to the level of disadvantage.” [1].

Francis-Oliviero and colleagues note that this definition leaves scope for broad interpretation, citing examples of single interventions with graded intensities; single interventions designed to disproportionately impact disadvantages groups; and the provision of different intervention for different groups [50]. Similarly, Benach and colleagues have argued that the essence of proportionate universalism is that “benefit increases through the gradient and the gap between socio-economic groups is reduced” [54]. However this definition and Marmot’s both leave room for inequalities to persist indefinitely, as long as they are continually narrowing. In contrast, ‘health for all’ seems to demand a closure of inequalities, manifest in the full realisation of health for every person.

## Application today

All UN member states have committed to achieving UHC by 2030 – guaranteeing access to quality essential health-care services for all [55]. This takes a Rawlsian input-based approach – guaranteeing that individuals receive comprehensive services but making no promises about the resultant distribution of health outcomes. No country has- or is likely to fully deliver UHC [56, 57] and gaping inequalities in life expectancy and other health outcomes remain within and between all countries [58–60]. As additional health services and financial protection schemes are rolled out, priority should be given to closing these unjust gaps. Proportionate universalism encourages health system leaders to deliver the greatest benefit for worst-off groups, whilst aiming to improve outcomes for all groups.

Any progress in this sphere is predicated on the collection and analysis of sociodemographic data so that managers can identify groups at the highest risk of being left behind. In their recent review, Francis-Oliviero et al. found very few examples or operational models that have successfully achieved proportionate universalism in service delivery [50]. More work is needed to develop and test routine approaches within healthcare.

Alongside this work, it is important to note that UHC focuses on service delivery rather than capabilities or seeking to influence unjust social norms and structures. We know that the social determinants of health are much more important in determining health outcomes than healthcare services, however the kind of whole-of-society ‘health in all policies’ approaches that grapple with underlying unjust social structures – central to the *Health For All by 2000* programme and the Alma-Ata and Astana Declarations—remain a fringe interest rather than a core priority for most people working in the field of health [61, 62]. Those of us who work on health inequalities should be seeking to influence the macro-level social structures that compound and perpetuate disadvantage, rather than simply tinkering with the health manifestations at the fringes.

The challenge of advancing UHC should be viewed primarily through a political lens, as it deals with power, influence, and the distribution of finite resources. In *Nicomachean Ethics* Aristotle argued that we should seek to participate in the political sphere and that politics is the higher form of ethics. This sentiment has been echoed by Ghilardi and colleagues who called for health workers and researchers become more politically and socially engaged as a core element of their work [63]. Virchow famously asserted that “medicine is a social science” whose practitioners are obligated to work with politicians in order to address the core drivers of ill health [64]. Many see political activism as lying beyond the purview of medicine [65]. Mahler acknowledged that the real work of advancing health for all is not a neat biomedical and managerial exercise, but a “complex, often messy process involving the interplay of physical, social, economic, and political variables” [13].

## Conclusion

WHO’s mandate of delivering health for all rests primarily on philosophical foundations; in an egalitarian belief that all humans have equal value, and that advancing care is a matter of justice. Whilst Mahler was alive to the prioritarian moral imperative driving the organisation’s work, seeking “a more equitable distribution of resources for health…in keeping with the principles of paying greater attention to the underprivileged” [40], the rationale underlying much of the WHO’s current work is framed in sufficientist, economic and technocratic terms. These appeals to nation enlightened self-interest reflect the prevailing nationalistic geopolitical zeitgeist, however WHO may gain additional traction in exploiting the philosophical foundations of its work, akin to the very successful rights-based calls for action on HIV [66].

Mahler used WHO’s mandate and voice to “focus world attention on health inequities” [67]. Framing UHC as a robust form of redistributive justice and putting more emphasis on the ethics of inaction may put additional pressure on politicians. WHO cannot escape the normative role that it plays, and should consider leaning into this space with the establishment of a ethics standing committee. There is precedent: an in-house ethicists was appointed in 1999 [68], and various task-and-finish consultive groups have been convened, including the aforementioned group for equity and UHC [43].

Approaches to delivering UHC are increasingly grounded in proportionate universalism, recognising that greater effort is required to optimise the health of marginalised groups. Whilst proportionate universalism is conceptually powerful, it has proven difficult to operationalise. There is a need for real-life models that provide graded levels of provision according to need. This will also translate into financing and provider payment systems that account for the effort involved in overcoming barriers to deliver care for marginalised groups.

An important first step is ensuring that our health systems adequately monitor and quantify the characteristics that are associated with poor outcomes. There are examples of nascent health service delivery approaches that aim to use such data to deliver proportionate universalism, but research is required to understand whether they achieve the stated aims of closing gaps whilst improving health outcomes for all. Finally, whilst it is vital that we develop health systems that account for and address inequalities, we must not fall into the trap of focusing wholly on downstream ‘cure’. We must seek to remedy unjust social structures through political engagement alongside targeted practical support.

## Data Availability

Not applicable.

## Abbreviations

UHC: Universal Health Coverage
WHO: World Health Organisation

## References

[1] Marmot M, Allen J, Goldblatt P, Boyce T, McNeish D, Grady M (2010). Fair Society Healthy Lives: The Marmot Review.

[2] Marmot M (2005). Social determinants of health inequalities. Lancet Lond Engl.

[3] World Health Organization. Health inequities and their causes. 2018. Available from: https://www.who.int/news-room/facts-in-pictures/detail/health-inequities-and-their-causes cited 2022 Mar 9

[4] NICE. Risk factors: Melanoma and pigmented lesions. 2017. Available from: https://cks.nice.org.uk/topics/melanoma-pigmented-lesions/background-information/risk-factors/ cited 2022 Jul 20

[5] Dwyer-Lindgren L, Kendrick P, Kelly YO, Sylte DO, Schmidt C, Blacker BF (2022). Life expectancy by county, race, and ethnicity in the USA, 2000–19: a systematic analysis of health disparities. Lancet.

[6] Hart JT (1971). The Inverse care law. Lancet.

[7] World Health Organization. Closing the gap in a generation: health equity through action on the social determinants of health - Final report of the commission on social determinants of health. Geneva; 2008. Available from: https://www.who.int/publications-detail-redirect/WHO-IER-CSDH-08.1 cited 2021 Nov 11

[8] WHO. Operational Framework for Primary Health Care. 2020. Available from: https://www.who.int/publications-detail-redirect/9789240017832 cited 2022 Mar 23

[9] WHO (1948). Constitution of the World Health Organization.

[10] WHO and UNICEF. Declaration of Alma-Ata. 1978. Available from: https://www.who.int/teams/social-determinants-of-health/declaration-of-alma-ata cited 2022 Mar 23

[11] WHO and UNICEF. Declaration of Astana on Primary Health Care. 2018. Available from: https://www.who.int/teams/primary-health-care/conference/declaration cited 2022 Mar 23

[12] Mahler H. Dr Halfdan Mahler’s address to the 61st World Health Assembly. 2008. Available from: https://www.who.int/director-general/speeches/detail/dr-halfdan-mahler-s-address-to-the-61st-world-health-assembly cited 2022 Sep 16

[13] Mahler H, Gunn SWA, Masellis M (2008). Health for All or Hell for All? The Role of Leadership in Health Equity. Concepts and Practice of Humanitarian Medicine.

[14] UN General Assembly. A/RES/75/310. Vision for Everyone: accelerating action to Achieve the Sustainable Development Goals. New York; 2021 Jul.

[15] Nozick R (1974). Anarchy, State, and Utopia.

[16] Mankiw NG (2013). Defending the One Percent. J Econ Perspect.

[17] Nietzsche FW (1969). On the genealogy of morals.

[18] Satz D, White S. What is wrong with inequality? In: Inequality: The IFS Deaton Review. The IFS; 2021. Available from: https://ifs.org.uk/publications/15639 cited 2021 Oct 13

[19] Aristotle. Nicomachean Ethics by Aristotle, translated by WD Ross. Oxford: Clarendon Press; 1925. Available from: http://classics.mit.edu/Aristotle/nicomachaen.html cited 2022 Sep 23

[20] The Meritocracy Trap by Daniel Markovits: 9780735222014 | PenguinRandomHouse.com: Books. PenguinRandomhouse.com. Available from: https://www.penguinrandomhouse.com/books/548174/the-meritocracy-trap-by-daniel-markovits/ cited 2022 Jul 20

[21] Nast C. “Tax the Rich” Protestors Descend on the Hamptons. Vanity Fair. 2022. Available from: https://www.vanityfair.com/style/2022/07/tax-the-rich-protestors-descend-on-the-hamptons cited 2022 Aug 11

[22] Frankfurt H (1987). Equality as a Moral Ideal. Ethics.

[23] World Bank. Measuring Poverty. World Bank. Available from: https://www.worldbank.org/en/topic/measuringpoverty cited 2022 Apr 14

[24] Smith A. An Inquiry Into the Nature and Causes of the Wealth of Nations. Legrand; 1776. 424 p.

[25] Ross RJS (2013). Bread and Roses: Women Workers and the Struggle for Dignity and Respect. Workingusa.

[26] Holtug N. Prioritarianism. Oxford Research Encyclopedia of Politics. 2017. Available from: 10.1093/acrefore/9780190228637.001.0001/acrefore-9780190228637-e-232 cited 2022 Aug 11

[27] Sen A. Development as Freedom. New York: Knopf; 1999. Available from: https://www.cambridge.org/core/journals/ethics-and-international-affairs/article/abs/development-as-freedom-amartya-sen-new-york-alfred-a-knopf-1999-380-pp-2750-cloth/B5758359AEF63D6FE020993160B5B3A6 cited 2022 Jul 21

[28] Dahlgren G, Whitehead M. Policies and strategies to promote social equity in health. Background document to WHO - Strategy paper for Europe. Arbetsrapport. Institute for Futures Studies; 1991 Dec. (Arbetsrapport). Report No.: 2007:14. Available from: https://ideas.repec.org/p/hhs/ifswps/2007_014.html cited 2022 Sep 16

[29] Rawls J. A Theory of Justice. Harvard University Press; 1971. Available from: https://www.jstor.org/stable/j.ctvjf9z6v cited 2022 Apr 14

[30] Rawls J (1993). Political Liberalism.

[31] Daniels N (2001). Justice, Health, and Health Care. Am J Bioeth AJOB.

[32] Daniels N, Kennedy B, Kawachi I. Social Justice is Good for Our Health. Boston Review; 2014. Available from: https://bostonreview.net/forum/norman-daniels-bruce-kennedy-ichiro-kawachi-justice-good-our-health/ cited 2022 Sep 16

[33] Ruger J (2004). Ethics of the Social Determinants of Health. Lancet.

[34] Ruger JP. Aristotelian Justice and Health Policy: Capability and Incompletely Theorized Agreements [PhD Thesis]. Dissertation; 1998.

[35] Sen A (1985). Commodities and Capabilities.

[36] Ruger JP (2008). Normative Foundations of Global Health Law. Georgetown Law J.

[37] Ruger JP (2004). Health and social justice. Lancet Lond Engl.

[38] Ruger JP (2007). Rethinking equal access: Agency, quality, and norms. Glob Public Health.

[39] Chan M (2016). Making Fair Choices on the Path to Universal Health Coverage. Health Syst Reform.

[40] Mahler H. Health for all by the year 2000. World Health. 1981;(February-March):3–5.

[41] World Health Organization. Universal health coverage (UHC). 2021. Available from: https://www.who.int/news-room/fact-sheets/detail/universal-health-coverage-(uhc) cited 2021 Nov 11

[42] Gwatkin DR, Ergo A (2011). Universal health coverage: friend or foe of health equity?. Lancet.

[43] World Health Organization. Making fair choices on the path to universal health coverage: final report of the WHO consultative group on equity and universal health coverage. World Health Organization; 2014. 78 p. Available from: https://apps.who.int/iris/handle/10665/112671 cited 2021 Oct 15 10.2471/BLT.14.139139 PMC4047814 24940009

[44] Summers LH (2015). Economists’ declaration on universal health coverage. Lancet.

[45] Thompson S, Hoggett P (1996). Universalism, selectivism and particularism: Towards a postmodern social policy. Crit Soc Policy.

[46] Anttonen: A, Häikiö L, Stefánsson K, Sipilä J. Universalism and the challenge of diversity. Cheltenham: Edward Elgar; 2012. 1–15 p. Available from: https://scholar.google.com/scholar_lookup?title=Universalism%20and%20the%20challenge%20of%20diversity&pages=1-15&publication_year=2012&author=Anttonen%2CA#d=gs_cit&u=%2Fscholar%3Fq%3Dinfo%3ANksq2_ow-YsJ%3Ascholar.google.com%2F%26output%3Dcite%26scirp%3D0%26hl%3Den cited 2022 Apr 1

[47] Carey G, Crammond B, De Leeuw E (2015). Towards health equity: a framework for the application of proportionate universalism. Int J Equity Health.

[48] Marshall T (1950). Citizenship and social class.

[49] Pratt A, Lavalette M. ‘Universalism or selectivism’. Oficial Policy: A Conceptual and Theoretical Introduction. London: Sage; 1997. 196–213 p. Available from: https://scholar.google.com/scholar_lookup?title=Universalism%20or%20selectivism%3F&pages=196-213&publication_year=1997&author=Pratt%2CA cited 2022 Apr 1

[50] Francis-Oliviero F, Cambon L, Wittwer J, Marmot M, Alla F (2020). Theoretical and practical challenges of proportionate universalism: a review. Rev Panam Salud Pública.

[51] Skocpol T. The Urban Underclass. Washington DC: Brookings Institution Press; 1991. Available from: https://www.brookings.edu/book/the-urban-underclass/ cited 2022 Apr 1

[52] Anand S. The Many Faces of Health Justice. London School of Economics International Inequalities Institute; 2021. 42. Available from: http://eprints.lse.ac.uk/112537/3/Final_Format_III_WPS_The_Many_Faces_of_Health_Justice_Anand.pdf

[53] Parfit D (1997). Equality and Priority. Ratio.

[54] Benach J, Malmusi D, Yasui Y, Martínez JM (2013). A new typology of policies to tackle health inequalities and scenarios of impact based on Rose’s population approach. J Epidemiol Community Health.

[55] UN General Assembly. A/RES/70/1: Transforming our world: the 2030 Agenda for Sustainable Development. 2015. Available from: https://www.un.org/ga/search/view_doc.asp?symbol=A/RES/70/1&Lang=E cited 2021 Nov 11

[56] World Bank. UHC service coverage index. 2022. Available from: https://data.worldbank.org/indicator/SH.UHC.SRVS.CV.XD?most_recent_value_desc=true&locations=GB-CA cited 2022 Jul 20

[57] Lozano R, Fullman N, Mumford JE, Knight M, Barthelemy CM, Abbafati C (2020). Measuring universal health coverage based on an index of effective coverage of health services in 204 countries and territories, 1990–2019: a systematic analysis for the Global Burden of Disease Study 2019. Lancet.

[58] WHO. GHE: Life expectancy and healthy life expectancy. 2022. Available from: https://www.who.int/data/gho/data/themes/mortality-and-global-health-estimates/ghe-life-expectancy-and-healthy-life-expectancy cited 2022 Jul 20

[59] Roser M, Ortiz-Ospina E, Ritchie H. Life Expectancy. Our World Data. 2013; Available from: https://ourworldindata.org/life-expectancy cited 2022 Jul 20

[60] Mackenbach JP, Valverde JR, Bopp M, Brønnum-Hansen H, Deboosere P, Kalediene R (2019). Determinants of inequalities in life expectancy: an international comparative study of eight risk factors. Lancet Public Health.

[61] Allen LN (2022). Primary health care is not just a service delivery platform. Lancet Glob Health.

[62] Allen LN, Smith RW, Simmons-Jones F, Roberts N, Honney R, Currie J (2020). Addressing social determinants of noncommunicable diseases in primary care: a systematic review. Bull World Health Organ.

[63] Ghilardi G, Campanozzi LL, Ciccozzi M, Ricci G, Tambone V (2020). The political nature of medicine. Lancet.

[64] (with acknowledgements to Siân Anis) JRA. Virchow misquoted, part‐quoted, and the real McCoy. J Epidemiol Community Health. 2006 Aug;60(8):671.

[65] Allen LN, Barry E, Gilbert C, Honney R, Turner-Moss E (2019). How to move from managing sick individuals to creating healthy communities. Br J Gen Pract.

[66] UNAIDS. Human rights-based approach to ending AIDS as a public health threat. 2022. Available from: https://www.unaids.org/en/topic/rights cited 2022 Sep 23

[67] Snyder A (2017). Halfdan Mahler. Lancet.

[68] Birmingham K (1999). WHO appoints first staff ethicist. Nat Med.

